# Non-state initiatives on enhancing counter-trafficking of Rohingya influx in Cox's Bazar of Bangladesh

**DOI:** 10.3389/fpubh.2023.1040546

**Published:** 2023-03-16

**Authors:** Edris Alam, Morshed Hossan Molla, Md. Kamrul Islam, Md. Arifur Rahman, Jishu Barua

**Affiliations:** ^1^Faculty of Resilience, Rabdan Academy, Abu Dhabi, United Arab Emirates; ^2^Department of Geography and Environmental Studies, University of Chittagong, Chittagong, Bangladesh; ^3^Disaster Action and Development Organisation (DADO), Chittagong, Bangladesh; ^4^Young Power in Social Action (YPSA), Chittagong, Bangladesh; ^5^Department of Civil and Environmental Engineering, College of Engineering, King Faisal University, Al-Ahsa, Saudi Arabia

**Keywords:** human trafficking, counter-trafficking, refugees, Rohingya, emergency response

## Abstract

Human trafficking is the third most lucrative form of trafficking in the world (following drugs and counterfeit goods). Multiple outbreaks of unrest between October 2016 and August 2017 in the Rakhine State of Myanmar triggered ~745,000 influxes of Rohingyas crossing into Bangladesh through the border boundaries at Teknaf and Ukhiya sub-districts of Cox's Bazar. In this regard, the media confirmed that over a thousand Rohingya people, particularly women and girls, were victims of human trafficking. This research aims to explore the underlying causes of human trafficking (HT) during emergency responses and seeks to understand how the knowledge and capacity of the refugee, local administration, and law enforcement agencies in Bangladesh can be improved in promoting counter-trafficking (CT) and safe migration processes. In order to achieve the objectives, this study reviews acts, rules, policies, and action plans of the Government of Bangladesh on the HT, CT, and safe migration processes. Then, a case study has been applied to present the ongoing CT and safe migration programs of an NGO called Young Power in Social Action (YPSA), which received funding and technical support from the International Organization of Migration (IOM) for this purpose. This study also evaluates the effectiveness of the program through conducting key informant interviews (KIIs) and focus group discussions (FGDs) with the beneficiary and non-beneficiary participants including refugees, law-enforcing agencies (LEAs), and NGOs in Teknaf and Ukhyia. Thus, this study identifies program-level strengths and weaknesses in relation to the CT and safe migration process and provides key directions on how they can be improved. It concludes that non-state actors have a significant role in preventing HT and promoting CT and safe migration for Rohingyas in Bangladesh.

## 1. Introduction

The Rohingya people have been experiencing massive state-driven discrimination, torture, and a sense of statelessness due to the deprivation of their basic needs and human rights in Rakhine State, Myanmar, since 1978 (1, 2). Due to such systematic persecution domestically, the Rohingya people fled to Bangladesh for five decades, with massive spikes following violent attacks in 1978, 1991–1992, 2016, and 2017 (3). Approximately 745,000 Rohingya refugees fled across the Bangladesh border from Rakhine state in Myanmar in August 2017, almost tripling the population of the Ukhiya and Teknaf sub-districts. By 21 June 2018, an estimated 745,000 Rohingyas had crossed into Bangladesh, joining nearly 212, 500 others who had arrived in earlier waves—in one of the largest population movements ever seen in such a short span. The vast majority of Rohingyas have been living in 34 congested camps in Ukhiya and Teknaf sub-districts (3). The largest camp, the Kutupalong–Balukhali Expansion Site, hosts ~626,500 refugees (3). Response by Bangladesh in sheltering the refugees has been widely acknowledged, but there were reports of communal violence, torture, abuse, and human trafficking (HT) of Rohingyas (4, 5).

The forcibly displaced Rohingya Muslim population who crossed the borders of Myanmar to Bangladesh traveled a laborious path by foot or boat for safety and were forced to leave everything behind. In fact, many of their houses were burnt down to ashes in the Rakhine state. Due to geographical location and the scarcity of economic activities where they took refuge, there has been evidence of HT for years (4). A number of national and international media reports documented HT in the Rohingya community both within and beyond Bangladesh. Rohingya girls and women suffer a higher risk of trafficking because they are the most vulnerable group. For example, the news outlet Al Jazeera on 3 December 2017 stated that Rohingya girls and women in Cox's Bazar are being sold as sex slaves. It is noted that 36,000 Rohingya orphan children whose parents were killed during the violence in Myanmar are potential victims of trafficking (VoT) (6). The situation report of the International Organization of Migration (IOM) noted 99 cases of HT, including 35 girls, 31 women, 8 boys, and 25 men (4).

To place the literature review, research methods, results, and conclusions into context, we have considered definitions used by the United Nations. According to Article 3, paragraph (a) of the protocol to prevent, suppress, and punish trafficking in persons recognized by the United Nations, “human trafficking” is defined as

“*the recruitment, transportation, transfer, harboring, or receipt of persons, by means of threat or the use of force or other forms of coercion; of abduction, of fraud, of deception, of the abuse of power or of a position of vulnerability, or of the giving or receiving of payments or benefits to achieve the consent of a person having control over another person, for the purpose of exploitation*” [(7), p. 42].

Exploitation includes “the exploitation of the prostitution of othersor other forms of sexual exploitation, forced labor or services, slavery or practices similar to slavery, servitude or the removal of organs” [(7), p. 42].

In order to create a holistic safeguard for the potential victims of trafficking, comprehensive counter-trafficking (CT) interventions including creating community awareness and mobilization; capacity-building within the community, its leaders, and law enforcement agencies (LEAs); and facilitating linkage and networking between VoTs and service providing entities were required (8, 9). Over the last four decades, the Government of Bangladesh (GOB) has been working with the IOM and the humanitarian community to meet the basic needs of the Rohingyas including safeguarding and protection, which was reinforced since August 2017. The Inter Sector Coordination Group (ISCG), led by the IOM, coordinated the Rohingya refugee crisis during the August 2017 influx and provided regular situation reports, updated settlement maps, and ensured effective response by humanitarian agencies.

Since the recent Rohingya influx in August 2017, donor agencies have been operating in the camps to provide protection, education, health, nutrition, food security, water, shelter, sanitation, and hygiene as well as to coordinate logistics for the sites. National and international NGOs have been working on HT in the Rohingya camp areas. Among them, YPSA, which is a leading, voluntary not-for-profit, and non-political organization, has been working on HT in Cox's Bazar since 1990. Donors contributed USD 2.59 billion between 25 August 2017 and 25 August 2021 to the Rohingya people (10). Notwithstanding the deficit of funding that was required for Rohingyas since the beginning of 2017 against an appeal of USD 434 million, the global community contributed USD 317 million as part of a 6-month response plan in 2018, and only USD 340 million has been pledged in 2021 against a request of USD 1,000 million (10).

If the planned activities are implemented, HT can be reduced to a great extent. YPSA in support of the IOM and other agencies is working to prevent HT and promote CT and safe migration processes for the Rohingya and host communities. Of these initiatives, a project entitled “initiatives to prevent human trafficking in emergency response” has been launched, aiming to increase the knowledge of refugees and host communities on CT, and safe migration capacity-building with the community leaders through ‘*key leader engagements*' (KLEs) liaising with NGOs and LEA to combat human trafficking.

This research aims to evaluate the effectiveness of the program through conducting interviews and focus group discussions (FGDs) with the beneficiary and non-beneficiary participants from school teachers, journalists, the Rohingya community, local administration, and LEA to combat human trafficking in one of the world's largest refugee camps and its adjacent areas.

## 2. Methods and approaches

### 2.1. Study area and location

**Figure 1:**
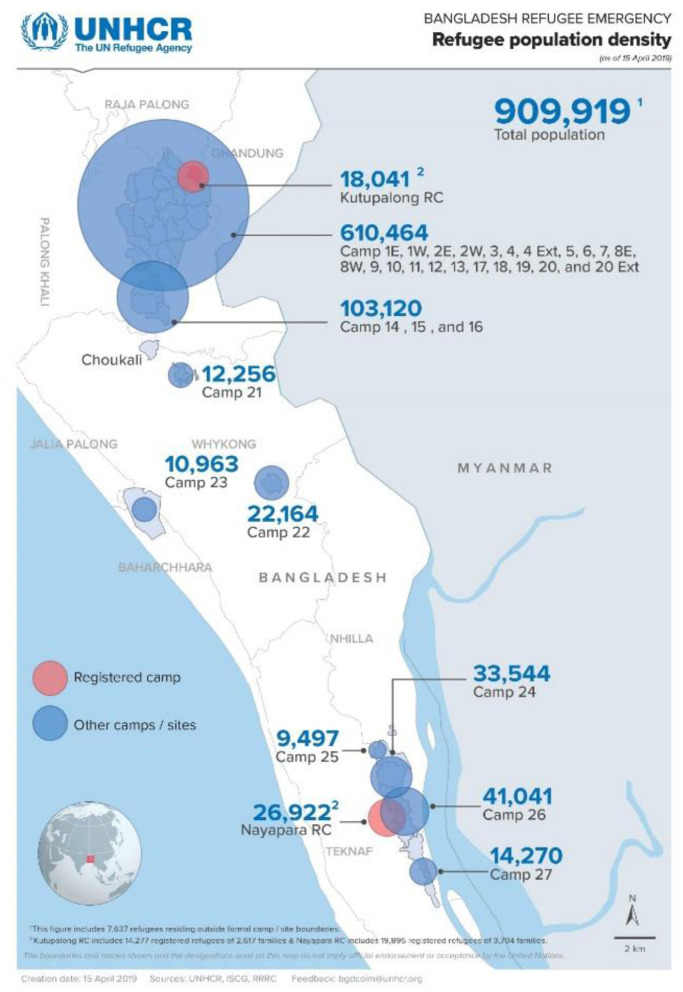
Refugee population distribution and camp locations [source: (19)].

The study was conducted on multi-stakeholders at two administrative sub-districts, Teknaf and Ukhia. Ukhia sub-district comprises Camp-−8E, 8W, 9, 10, 11, 12 13, 14, 15, 16, 17, 18, 19, and 20, and Teknaf sub-district involves *Leda* (the name of geographical unit) MS, Leda A, Leda-B, Leda C, and Leda D (Figure 1).

### 2.2. Research design

The study followed a mixed-method approach of using both qualitative and quantitative methods that exacerbate benefits when they are applied separately (11). A number of authors such as Islam and Walkerden (12) and Islam and Hasan (13) have used a mixed-method approach for similar studies. For the convenience and effective conduction of this research, it has been implemented into two major phases: (1) evaluation of the project activities and (2) evaluation of the effectiveness of the program through conducting key informant interviews (KIIs) and focus group discussions (FGDs) with the beneficiary and non-beneficiary participants.

In the first phase, this study conducted a documentary review of the project activities, relevance, and effectiveness of the project strategy, project management arrangement and intervention reports, training manuals, and quarterly progress reports on HT, CT, and safe migration processes implemented by YPSA that received funding and technical supports from the International Organization of Migration (IOM) for this purpose. In addition, the evaluation team conducted dedicated meetings and conversations with project staff on different aspects of project management and interventions. Finally, an evaluation of the effectiveness of the program is provided which was a result of conducting KIIs and FGDs with both beneficiary and non-beneficiary participants from teachers, journalists, Rohingya refugees, local administration, and LEAs in Teknaf, and Ukhyia sub-districts.

### 2.3. Participant selection, questionnaire, data collection, and analysis

In the second phase, the study conducted a cross-sectional study interviewing both beneficiaries and non-beneficiaries with a pre-tested questionnaire that consists of both qualitative and quantitative questions. The questionnaire was pre-tested on 10 beneficiaries comprising five members from each group. The respondent's comments were considered while developing the main evaluation questionnaire. Targeted sampling was used to collect data from Rohingya and non-Rohingya communities between September and October 2019. Prior to conducting interviews, the participants were briefed on ethical protocols, the research purpose, and possible outcomes. Next, after being informed of the earlier, they could decide to either proceed and participate in the interviews or turn down the invitation. For this study, ethical clearance was sought and approved by the University of Chittagong's Ethics Committee for the Department of Geography (Protocol number: CUNI-13/0022). No personal identifiers were collected. The participants were coded by beneficiaries and non-beneficiaries with their professional identities remaining anonymous. Thus, the study maintained ethical standards, specifically in accordance with the Declaration of Helsinki—ethical principles for research involving human subjects.

In this study, experimental research was designed in order to understand the knowledge gap between the beneficiary and non-beneficiary groups after project implementation. The data were collected using information from the questionnaire which assessed knowledge and awareness differences about HT, CT, rules and regulations, and the safe migration process. The participants included school teachers, journalists, Rohingya households, and *Majhi*s (*Majhis* are community leaders of the Rohingyas).

Before conducting interviews, a checklist was prepared for the beneficiary participants who would be invited for interviews and FGDs. A total number of 48 interviews were conducted with the beneficiary and non-beneficiary groups to understand the difference in knowledge between the groups during the mid-term evaluation of the project. The interviews were conducted with school teachers and journalists.

**Figure 2:**
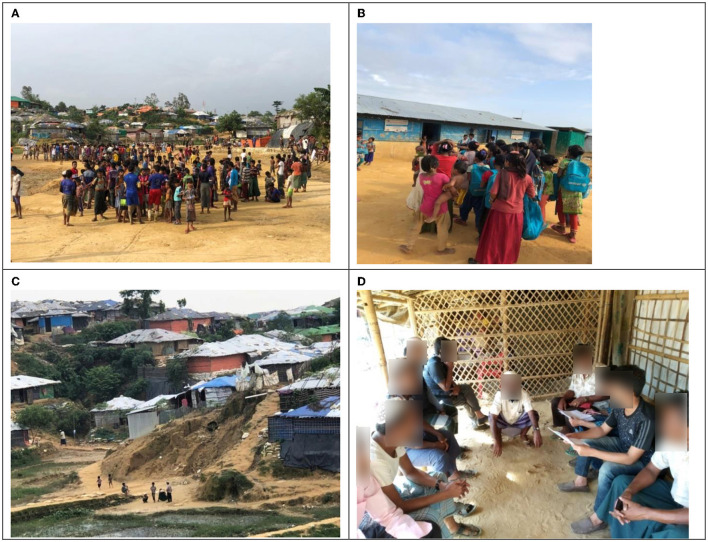
Rohingya camp at Kutu Palong: **(A)** adolescent boys gathering in a lower valley area for a football match, **(B)** adolescent girls in front of a temporary school, **(C)** settlement in camp, and **(D)** focus group discussion (FGD) in the camp (source: Field survey).

Focus group discussions were conducted with the beneficiary and non-beneficiary *Majhis* because of their strong influence on the Rohingya community. Of the four FGDs conducted, two each were conducted with beneficiary *Majhi* and non-beneficiary Majhi, respectively. The time and location of FGDs were pre-arranged in consultation with the participating Majhis. Eight *Majhis* who were cooperative and enthusiastic attended each FGD session. *Majhis* who participated in the FGDs are male subjects who represent muscular dominancy in the group. Finally, observation (overt and covert) has been used to document eyewitnesses and to understand the ongoing project interventions by project staff. This field visit also helps to understand the camp entry and exit gates, connectivity to adjacent roads and host communities, and overall environment (Figure 2).

Data collected through interviews and FGDs were carefully transcribed and checked immediately after returning from the field with the data collectors transcribing verbatim the recorded speech of their respondents. After that, relevant data were analyzed with narrative analysis, where researchers interpret stories that are told within the context of HT, CT, and the migration process. A triangulation data analysis technique was used to examine qualitative and quantitative data based on a thematic approach. This integrated and triangulated analysis ensured that the study could find interactions and influence of variables from different dimensions.

Quantitative data on the Likert scale were entered into SPSS-25 IBM. We assessed the level of understanding by both beneficiary and non-beneficiary participants about HT, CT, and safe migration processes on a 5-point Likert scale, ranging from (5) very confident to (1) very low confident (14). The test has been able to explore the strong significant relationship among the variables (*p*< *0.01*). The weightage analysis method was used for exploring the quantitative finding of the study. Cronbach's alpha statistics were used to check the reliability of the weightage analysis performed. Cronbach's alpha reliability coefficient normally ranges between 0 and 1. The closer the coefficient is to 1.0, the greater the internal consistency of the items (variables) in the scale. Cronbach's alpha coefficient increases either as the number of items (variables) increases, or as the average inter-item correlations increase (i.e., when the number of items remains constant). After the completed Cronbach's alpha statistic to test reliability, the test found excellent condition (0.9) out of the five levels (15).

## 3. Results

The results of the study are presented in two sections. First, we present the results of a review of the evaluation of the humanitarian initiatives led by YPSA. Especifically, we reviewed project activities such as intervention reports, quarterly progress reports, and training manuals. Second, the paper presents the research findings from the evaluation with the beneficiary and non-beneficiary stakeholders.

### 3.1. Project interventions

The project has been implemented in three different categories in 21 areas comprising 14 camps, five *Leda* (specific name of the camp), and two locations in two administrative areas, particularly the Ukhia and Teknaf sub-district. Over 463,515 individuals live across 21 areas. The project's staff have attempted to implement different trafficking prevention activities for targeted beneficiaries. These programs directly addressed four different activities, namely courtyard meetings, drama/cultural events on CT, meetings with *Majhi*, and CT messages on Radio Naf (16).

#### 3.1.1. Program on CT issues and referral mechanisms with Majhi

The project staff arranged the orientation program about CT issues and referral mechanisms with *Majhi* at the camps in Ukhiya and Teknaf. During these orientation programs, the project staff clearly discussed project objectives and activities. First, the implementation team has emphasized on concept, causes, consequences, and prevention of HT. Second, they highlighted the present situation of HT, potential victims, traffickers, prevention measures, and related unlawful incidences such as drug-trafficking and gender-based violence (GBV) such as early marriage and polygamy which are considered as the pull factors to HT. Finally, they explained how to deliver referral pathways and how services were made available for victims. The trainers who have expertise in HT have facilitated these programs at camp areas in the presence of YPSA's executive members.

#### 3.1.2. The capacity-building of teachers, camp focal person, and host community

In the past, YPSA was partnered with the IOM on a project entitled “Initiatives to Prevent Human Trafficking in Emergency Response (PHTER)” to increase the knowledge of refugees and host communities on trafficking and safe migration, and capacity-building of community leaders, NGOs and LEA to combat HT. The participants in the capacity-building program receive training on the causes and consequences of HT, how to work together for the prevention of HT, the identification of the victims, and the referral mechanisms to learn about the key laws such as The Prevention and Suppression of Human Trafficking Act 2012 (16). In this program, positioning paper, power-point presentation, concept note, video clips, and video documentary about HT and CT have been displayed.

#### 3.1.3. The awareness-related modules for Rohingya and host community

The project team has prepared the module on developing awareness and capacity-building of Rohingya, Majhi, camp focal point person, and host community in selected camps areas. First, the module focused on the concept, causes and consequences, and prevention of HT. It also highlights the safe migration process. Then, it referred to a different awareness and prevention-related initiatives, particularly courtyard meetings, introducing District Manpower Office (DEMO), HT-related reference mechanisms, and provision of laws and policies. Finally, it developed and implemented referral modules to prevent HT and promote CT and safe migration.

#### 3.1.4. The quarterly progress reports

The quarterly reports are prepared to monitor and evaluate project activities and deliverables. Each activity was completed on time, except the activity on the orientation of the HT Act and referral services with law enforcement agencies. It was noted that a number of factors including prolonged rainy season and associated flash flooding, late approval from the office of the Refugee Relief and Repatriation Commissioner (RRRC), and staff dropouts interrupted the program's activities.

The quarterly progress report has evidenced that the project staff have successfully developed awareness of HT- and CT-related modules and arranged 216 courtyards meeting out of a total of 770 meetings with the Rohingya and host community. Some of the courtyard meetings were interrupted due to bad weather conditions. A session titled “Orientation on Safe Migration Related issues for potential and out-going migrants” was facilitated for the host community at DEMO, Cox's Bazar. The project organized five meetings with *Majhis* and host communities on CT issues and referral mechanisms. They organized a “Workshop for Media Professionals/Journalists on Human Trafficking Act & Preventing TIP” at the Mushroom Center, Bus Terminal, and Cox's Bazar Sadr. Moreover, the project staff observed “World Day Against Trafficking-in-Persons” and organized four street dramas to create awareness about anti-human trafficking in the Rohingya and host community. Furthermore, the project launched an awareness program on HT through community Radio Naf 99.2 FM which is a popular media in the project intervention area. Therefore, after the first quarter, project staff developed and maintained effective communication channels with targeted stakeholders.

### 3.2. Results from face-to-face interviews

#### 3.2.1. Results from the school teachers and journalists

The findings suggest that school teachers and journalists who received orientation have higher levels of awareness and knowledge about the HT act and other acts, as well as referral services and safe migration, than non-beneficiary/non-beneficiaries. They have gained knowledge or ideas about the Prevention and Suppression of Human Trafficking Act 2012. In contrast, the non-beneficiary participants were very unaware of the act. All participants in KIIs have an understanding of the first chapter of the Prevention and Suppression of Human Trafficking Act 2012. The beneficiary group could mention ways to provide referral services for HT. The majority of participants were confident in explaining the HT act and referral services in both camp areas and host communities. On contrary, non-beneficiary participants were less confident in explaining about HT act and referral services in both camp areas and host communities. The beneficiary participants could identify nine types of acts and rules related to HT: (1) Bangladesh Constitution (Articles, 34, 38); (2) Prevention and Suppression of Human Trafficking Act 2012; (3) Women and child prevention and suppression SAARC convention 2012; (4) Child Act 1974; (5) Bangladesh Labor Act 2006; (6) Women policy 2011; (7) Child right policy 2011; (8) Immigration rules 1982; and (9) Bangladesh Passport Act 1973.

All beneficiary participants knew about the Bangladesh Constitution Articles, 34 and 38 on trafficking issues, Prevention and Suppression of Human Trafficking Act 2012, Child Act 1974, Women policy 2011, and Child right policy 2011. The majority of non-beneficiary participants personally knew about Bangladesh Constitution (Articles, 34, 38), Child Act 1974, and the Women policy 2011. Beneficiary participants suggested that they benefitted and received different training activities (i.e., orientation sessions) organized by YPSA on the human trafficking act and referral services. By attending orientation sessions and training, the participants have a clear idea about trafficking processes and traffickers, knowledge about anti-human trafficking tribunals and the trial of offenses, the human trafficking Act and rules, and how and where victims get security and legal support. The findings from KIIs have identified two major weaknesses: (1) training session on HT and CT is short; (2) there were not adequate local district and sub-district levels civil administrators, LEA (i.e., Bangladesh police, Boarder Guard of Bangladesh (BGB) and lawyers in the process.

#### 3.2.2. Findings from the Rohingya community

In response to causes of HT, most of the beneficiary participants identified nine reasons that include (1) financial insolvency; (2) expectation of a good job; (3) greed for money; (4) unemployment; (5) lack of awareness; (6) uncertain future; (7) violence; (8) lack of job; and (9) lack of appropriate housing. There are two types of factors, push and pull, which are responsible for human trafficking. During our opinion survey, beneficiary participants mentioned that 11 types of push factors responsible for the irregular migration include: (1) improved life expectation; (2) unemployment; (3) family feud; (4) a high number of forced marriages; (5) more earning offer; (6) border crossing opportunity; (7) early marriage; (8) physical, mental and sexual harassment; (9) helpless and dowry; (10) experiencing conflict situation; and (11) uncertain life and violence. Of these factors, unemployment, more earning offer, improved life expectation, and border crossing opportunity were the most prevalent push factors for HT among Rohingya communities.

Beneficiary participants identified that six types of traffickers are involved in their regular migration processes. This includes relatives, neighbors, friends, influencing local leaders, employers/persons who help them to get employment, and unknown persons. The non-beneficiary participants said that five types of traffickers are responsible for illegal migration such as relatives, neighbors, friends, employer/person helping them to get employment, and unknown person, respectively. However, both beneficiary and non-beneficiary groups opined that most people are trafficked by strangers. In response to knowledge about victims of trafficking, both beneficiary and non-beneficiary Rohingya communities have clear ideas about who are the potential victims of HT. The potential victims include children, adolescents, males, females, widows, isolated persons, homeless persons, unemployed persons, and orphans.

By attending CT and safe migration-related orientations such as courtyard meetings, day observation, Radio Naf, and street drama, beneficiary participants have a better understanding of counter-trafficking and safe migration than non-beneficiary participants do. The beneficiary Rohingya communities confirm that they are aware of six types of incidences involved with HT such as threat, force, kidnapping, exploitation, taking disadvantaged situations, and offering money. The participants heard about the incidence of threats, force, and kidnapping in the areas. They also reported that the victims were psychologically stigmatized and experienced different types of consequences such as personal (i.e., an item to be sold out for commercial purposes), health (i.e., physical injury and mental depression), bad reputation, isolation or compel to leave overseas, social and economic losses.

The beneficiary host community participants could identify a series of steps for safe migration that were introduced in training and courtyard meetings. These include having a passport, Bureau of Manpower Employment and Training (BMET) registration, registered recruitment agency, paying money with receipt, legal job agreement, visa, and its verification processes, BMET release letter and smartcard, and opening a bank account, respectively. In contrast, non-beneficiary participants opined that having a passport, visa, and its verification processes and BMET registration are the main steps for safe migration. Beneficiary participants suggested that courtyard meetings, street drama, day observation, and awareness of HT through Radio Naf are the most effective ways for reducing human trafficking and promoting CT in these areas. In response to queries relating to referral services for victims in the camp, the beneficiary participants mentioned three types of referral services: (1) referring to the relevant organization/entities for counseling and basic support; (2) referring to the relevant organization and entities for legal help and; (3) proper maintenance for the referral system.

This evaluation has attempted to identify the weakness of CT and safe migration-related project items and delivery mechanisms. The participants identified six major weaknesses that include: (1) the number of meetings is less; (2) the duration of meetings was short; (3) the presence of women is less; (4) long time gap between two meetings; (5) instead of using *Chitttagonian* (local language) language, the Rohingya communities suggest to use their own Rohingya language in Radio Naf; and (6) excessive people in each meeting.

#### 3.2.3. Results from Majhi

Majhis from both beneficiary and non-beneficiary suggested that eight types of victims are trafficked, due to irregular migration in these areas. These victims are children, adolescents, males, females, widows, isolated, homeless persons, unemployed persons, and orphans. More recently both children and women have been trafficked by strangers or unknown persons and friends in these areas. By attending CT and safe migration-related awareness training, meetings, street drama, and listening to Radio Naf programs, the beneficiary Majhis could more confidently explain the legal procedure on CT and referral services for victims of trafficking. They also could confidently explain more about the push and pull factors of trafficking, potential type of traffickers, the most vulnerable victims, and safe migration procedures.

#### 3.2.4. Quantitative evaluation of beneficiaries' understanding of CT

Beneficiaries from both Rohingya and host communities became aware of HT and CT because they enhanced their understanding from different types of awareness programs such as courtyard meetings, street drama, day observation, and programs on HT through Radio Naf. Quantitative analysis suggests that the beneficiary participants from school teachers and journalists had a better understanding of HT, CT, and safe migration than the remaining three groups (Table 1). This may be due to their prior knowledge gained through education and professional experience. In contrast, the participants from the Majhi showed comparatively less understanding of HT, CT, and safe migration because of their limited education and level of prior knowledge.

**Table 1:** Understanding and interpreting of beneficiary participants about CT and safe migration (*N* = 12).

**List of item**	**5**	**4**	**3**	**2**	**1**	**Total calculated weight**	**Mean score**	**Average score**	**St. deviation**
Journalists and school teachers	5	5	2	0	0	51	4.2	3.6	0.36
Rohingya community	4	3	1	2	2	41	3.4		
Host community	4	3	2	1	2	42	3.5		
Majhi	3	4	3	2	0	42	3.5		

N.B: very low confident = 1, low confident = 2, neutral = 3, confident = 4, and very confident = 5.

The weighted analysis explored that the mean score of school teachers and journalists is high (4.2) among the four groups of participants. The average score is 3.6. The score represented a strong understanding among beneficiary stakeholders about CT and safe migration-related issues in the area. The standard deviation is 0.36, implying that most of the numbers are very close to the average. This means that the project interventions have successfully helped to understand and interpret the causes of CT and safe migration from different stakeholders.

#### 3.2.5. Quantitative evaluation of non-beneficiaries understanding of CT

The weighted analysis suggests that the mean score of Majhi is low (2.0) among the four non-beneficiary groups (Table 2). The average score of the non-beneficiary group is 2.5 which is lower than beneficiary groups. The standard deviation is 0.41 which is also higher than the beneficiary group. Thus, qualitative and quantitative data are consistent about the effectiveness of the project intervention activities and the necessity to expand such activities among the wider communities.

**Table 2:** Understanding and interpreting of non-beneficiary participants about CT (*N* = 12).

**List of item**	**5**	**4**	**3**	**2**	**1**	**Total calculated weight**	**Mean score**	**Average score**	**St. deviation**
Journalists and school teachers	2	1	3	3	3	32	2.6	2.5	0.41
Rohingya displaced people	1	3	2	3	3	32	2.6		
Host community	0	4	4	4	0	36	3.00		
Majhi	0	1	3	4	4	25	2.00		

N.B: very low confident = 1, low confident = 2, neutral = 3, confident = 4, and very confident = 5.

#### 3.2.6. The valuation of HT and CT interventions and the scope for improvement

The quantitative evaluation of the HT and CT intervention activities undertaken by the YPSA suggests four types of activities including orientations on safe migration with potential migrants, day observation, street dramas on CT, and awareness on CT through Radio Naf are found useful. The other four types of activities, courtyard meetings, orientation sessions with Majhis, orientations on the human trafficking act and referral services, and capacity-building workshops for journalists are useful. The participants show positive gestures to attend the activities in the future and also provide specific suggestions for each type of activity (Table 3).

**Table 3:** Summary findings on the assessment of the project intervention activities of HT and CT.

**Activities on CT**	**Evaluation by the participants**	**Participation in future**	**Suggestions for improvement**
Court-yard meetings	Useful	68%	Direct stories from victims from Rohingya communities. More frequent meetings
Orientations on safe migration with potential migrants	Very useful	65%	A triangular partnership among IOM, YPSA, and DEMO on CT and safe migration. Direct participation of DEMO senior officials
Day observation	Very useful	74%	Current efforts are satisfactory
Street dramas on CT	Very useful	82%	Frequent street dramas
Awareness on human trafficking through Radio Naf	Very useful	84%	The beneficiary participants wanted to disseminate awareness activities through Radio Naf *via* the Rohingya language. Stories directly from Rohingya victims of human trafficking should be disseminated
Orientation sessions with Majhis	Useful	63%	Frequent sessions incorporating more Majhis
Orientations on the human trafficking act and referral services	Useful	59%	More teachers and journalists should be covered under this program. LEA was not included in the previous sessions and they should be involved at an early stage.
Capacity-building workshops for media professionals/journalists	Useful	61%	Current activities should be continued by extending to more media professionals.

## 4. Discussion

By conducting a narrative review of the literature, Lugova et al. (17) reported that the humanitarian crisis in the Democratic Republic of the Congo has exacerbated sexual and gender-based violence, including HT experienced by women and young girls in refugee settings. On occasion, national and transnational traffickers target economic and political migrants because of their disadvantaged circumstances in Tunisia (18). The HT among the Rohingya community in post-conflict settings reported an increasing trend due to a lack of basic needs in camp areas since 2017.

Counter-trafficking interventions gained phenomenal popularity since the adoption of the UN Trafficking Protocol 2000 which prompted the undertaking of a variety of efforts from the UN agencies, national governments, International Non-Governmental Organizations (INGOs), and NGOs (8). The activities to address HT includes strengthening legal-institutional means, community-led counter-trafficking at the field level, and providing support for those exploited. Our efforts in this study are mostly related to identifying pull factors of trafficking at the community level and how to promote CT and safe migration. Thus, the valuation of the project-level activities identified a few crucial loopholes in humanitarian interventions and the performance of non-state actors in Rohingya camps and adjacent host community areas (Table 4). The study demonstrates the requirement for implementing field-level interventions by documenting knowledge differences between the beneficiary and non-beneficiary groups.

**Table 4:** Ways to improve interventions to stop HT and enhance CT.

**Details of recommended activity**	**Suggested by**	**Implementing stakeholders**
Frequent meetings to encourage and build awareness to stop falling into the grip of the broker	Rohingya refugee and host community	Partnership with community, CBOs, and NGOs
More street dramas since they are very effective for awareness generation and community engagement	Rohingya refugees and host community	Partnership with community and NGOs
Engaging more women participants for each event	Rohingya refugees, Majhis, teachers, journalists, and host community	Partnership with community and NGOs
Education and other training activities launched by the other stakeholders at the camp may include HT awareness and CT process	School teachers and journalists	All NGOs and international donor agencies
HT and CT initiatives should be facilitated in the Rohingya language	Rohingya refugee	Radio Naf by NGO
Better meeting space providing perfect sound system to reach all participants	Rohingya refugees	NGOs
The duration of the court-yard meeting should be longer allowing the moderator to complete all awareness-related activities	Rohingya refugees	NGOs
A local community leader is to be considered an actor or factor for every initiative.	Rohingya refugees and host community	Partnership with community, NGOs, and local administration
LEA and their inclusion are helpful for awareness generation and peace keeping	Rohingya refugees, Majhis, teachers, journalists, and host community	NGOs and local administration
Surveillance of LEA with modern equipment at entry and exit gates at camp to identify victims of trafficking.	Rohingya refugees, Majhis, teachers, journalists, and host community	The central government and local administration
Considering feedback from participants about the program and expectations from future events.	School teachers and journalists	NGOs
Enhancing partnerships with the community, CBOs, NGOs, and local administration especially police for the CT process	School teachers and journalists	Partnership with the community, CBOs, NGOs, and local administration
The execution of exemplary punishment for traffickers and dissemination of prosecution through the media	School teachers and journalists	Local civil administration and Police
Awareness building for a safe migration process	School teachers and journalists	Partnership with community, NGOs, and local administration
Engaging local influential persons, political and social leaders in CT and safe migration activities	School teachers and journalists	CBOs, NGOs, and local administration
Ensuring sufficient food for the Rohingya people	Majhi	NGOs and local administration
Not allowing unknown people in camp without holding appropriate identity	Majhi	Local administration particularly law enforcing agencies

The key project intervention activities, courtyard meetings, orientations on safe migration with potential migrants, day observation, and street dramas on CT were attended by the beneficiary participants. This study identifies several key loopholes that need to be treated and improved. Interventions are required for future project deliveries. First, with regard to awareness generation activities, the beneficiary participants support ongoing courtyard meetings, orientation sessions, and street dramas that need to occur more frequently in a better place with appropriate facilities. Although women participated in each event, their equal participation should be ensured by the implementing organizations. Education and training activities launched by the other organizations at the camp may include HT awareness and the CT process. Awareness activities should be delivered in the local dialect especially when targeted participants are Rohingya communities. Because local administration and law enforcement authorities play a crucial role in the CT process, their inclusion should be one that will enhance the community's confidence in such types of interventions. Local community leaders and *Majhi* are to be empowered considering their influence and trust in the communities. It will require increasing the surveillance of LEA with modern equipment at each entry and exit gate. Sufficient food for the Rohingya people must be ensured because food insecurity is the main push factor to fall victim to traffickers. Partnership development with community, community-based organizations (CBOs), NGOs, and local administration, especially police for the CT process will be of great help.

## 5. Conclusion

The Government of Bangladesh and Bangladeshi communities showed great humanity by hosting over 900,000 stateless Rohingyas which is appreciated by international communities. It was a significant challenge given the limited time to organize all the required food, clothing, shelter, and arrange for health opportunities for the large influx of Rohingyas. Due to limited basic needs and untight security options, there was evidence of HT of Rohingya refugees. Several international agencies, particularly the IOM, in partnerships with NGOs were active since August 2017 providing support to the local government and Rohingya community to promote CT activities. The activities implemented by the YPSA in the camp and adjacent host community demonstrated their effectiveness in creating awareness about HT, promoting CT, and safe migration in the area. The findings of this research and recommendations may be useful to enhance CT and safe migration in the refugee and non-refugee setting in Bangladesh and beyond. The study mainly evaluates HT-related activities implemented at the field level and the effectiveness of their deliveries. Thus, comments cannot be made on the actual outcome of the project activities. Therefore, more studies need to be conducted to evaluate the impact of the CT programs that were implemented following a rapid and large influx of Rohingya refugees in Bangladesh.

## Data availability statement

The original contributions presented in the study are included in the article/supplementary material, further inquiries can be directed to the corresponding author.

## Ethics statement

The studies involving human participants were reviewed and approved by the Institutional Review Board (or Ethics Committee) of the Department of Geography and Environmental Studies at the University of Chittagong, Bangladesh (protocol code: CUNI-19/005 and date of approval: 01 September 2019) for studies involving humans. Written informed consent was obtained from the individual participant for the publication of any potentially identifiable images or data included in this article. The patients/participants provided their written informed consent to participate in this study.

## Author contributions

Conceptualization and writing—review and editing: EA, MAR, and JB. Methodology and formal analysis: EA and MHM. Validation: EA, MKI, and MAR. Investigation: EA, MHM, and JB. Resources and supervision: EA and MAR. Data curation and visualization: EA and MKI. Writing—original draft preparation: EA and MHM. Project administration: EA.
